# Unveiling Novel Structural Biomarkers for the Diagnosis of Glaucoma

**DOI:** 10.3390/biomedicines12061211

**Published:** 2024-05-29

**Authors:** Yu-Chien Tsai, Hsin-Pei Lee, Ta-Hsin Tsung, Yi-Hao Chen, Da-Wen Lu

**Affiliations:** 1 Department of Ophthalmology, Tri-Service General Hospital, National Defense Medical Center, Taipei 114, Taiwan; 2 Department of Ophthalmology, Taoyuan Armed Forces General Hospital, Taoyuan 325, Taiwan

**Keywords:** diagnosis, structural, image, biomarkers, glaucoma

## Abstract

Glaucoma, a leading cause of irreversible blindness, poses a significant global health burden. Early detection is crucial for effective management and prevention of vision loss. This study presents a collection of novel structural biomarkers in glaucoma diagnosis. By employing advanced imaging techniques and data analysis algorithms, we now can recognize indicators of glaucomatous progression. Many research studies have revealed a correlation between the structural changes in the eye or brain, particularly in the optic nerve head and retinal nerve fiber layer, and the progression of glaucoma. These biomarkers demonstrate value in distinguishing glaucomatous eyes from healthy ones, even in the early stages of the disease. By facilitating timely detection and monitoring, they hold the potential to mitigate vision impairment and improve patient outcomes. This study marks an advancement in the field of glaucoma, offering a promising avenue for enhancing the diagnosis and possible management.

## 1. Introduction

Glaucoma stands as a pivotal concern in ophthalmology and is renowned for being a leading cause of irreversible blindness globally. It impacted an estimated 80 million people worldwide in 2020 and is still increasing till now, imposing a tremendous financial burden on both the individual and society [1,2]. It is a group of chronic and progressive retinal and optic neuropathies, characterized by irreversible morphological changes at the optic nerve head (ONH) and the inner retinal layers and visual field defects, which are associated with retinal ganglion cell (RGC) loss and an elevation of intraocular pressure (IOP) [3]. Glaucoma may not have any symptoms in the early stages and the optic nerve injury is already quite advanced when the patient presents with initial visual field defects, which makes it “the silent thief of sight” [4]. In most cases, the central vision is the last to be affected. As a consequence, glaucoma may remain undetected until it reaches a moderate or severe stage, thereby causing treatment and diagnosis delays and resulting in unaltered visual acuity change. A person may develop a heightened propensity for incidents involving falls and collisions with objects while walking, as well as encounter challenges while driving, by that juncture. This underscores the critical importance of early detection and vigilant monitoring to thwart visual disability associated with this condition. In this pursuit, the exploration and identification of glaucoma biomarkers have opened new horizons for early diagnosis, understanding risk profiles, pinpointing damage progression, and monitoring treatment response, with a hope of revolutionizing glaucoma management [5].

Diving deeper, the article unfolds the role of structural biomarkers in enhancing the specificity and sensitivity of glaucoma diagnosis [5]. It proposes innovative methodologies such as optical coherence tomography (OCT) and magnetic resonance imaging (MRI) in glaucoma research, showcasing how these methods complement traditional diagnostic approaches to offer a fuller, more accurate picture of the disease [6,7]. While elucidating the challenges faced and proposing future directions, this narrative is set against the backdrop of the newest findings in structural biomarker research, promising to significantly advance the field of glaucoma diagnosis [5,7].

## 2. Traditional Diagnostic Methods

Diagnosing glaucoma involves a comprehensive assessment of various ocular parameters to identify signs of optic nerve damage and associated vision loss [8]. The initial step often includes measuring intraocular pressure (IOP) through tonometry, as elevated IOP is a primary risk factor for glaucoma. However, the IOP is often influenced by corneal properties such as corneal thickness, corneal curvature, and elastic properties; thus, additional examination of the cornea or using specific equations for modified-IOP calculation is very important [9,10]. Among the glaucoma patients, almost one-third of the eyes have normal IOP, underscoring the necessity of conducting further diagnostic imaging instead of relying on the IOP readings [11]. Fundus photography allows for visualization of the optic nerve head and adjacent tissue, enabling clinicians to detect structural abnormalities indicative of glaucomatous damage like enlargement of the cup, disc hemorrhages, pallor of the disc, neuroretinal rim thinning, and neovascularization. OCT uses laser beams to provide high-resolution images in evaluating the ocular structures including the thickness of the retinal nerve fiber layer and neuroretinal rim of the optic nerve head [12]. Perimetry assesses peripheral vision and aids in identifying the characteristic patterns of visual field loss associated with glaucoma such as nasal step, temporal wedge defect, classic arcuate defect, generalized constriction, or tunnel vision defect with temporal crescent sparing [13]. Additionally, gonioscopy assesses the drainage angle of the eye, which is crucial in determining the risk of angle-closure glaucoma, and pachymetry for measuring central cornea thickness plays an important role in glaucoma diagnosis [14]. 

However, the limitation of current methods for diagnosing glaucoma is the incapacity to definitively diagnose the condition prior to significant glaucomatous damage. While RGC apoptosis has been recognized as the initial stage of cellular demise in glaucoma, it is approximated that a significant proportion of RGCs are lost prior to the detection of visual field abnormalities by conventional clinical examinations. The progress and incorporation of many diagnostic methods, such as imaging technology and functional tests, improve the accuracy of early glaucoma diagnosis and allow prompt management to maintain visual function [15].

## 3. Emerging Structural Diagnostic Biomarkers

In the quest for early and accurate diagnosis of glaucoma, structural biomarkers have emerged as a pivotal area of research. These biomarkers offer a promising avenue for detecting glaucoma at its nascent stage, potentially revolutionizing the approach to managing this vision-threatening condition. In diagnosing and tracking the progression of glaucoma, direct examination of the optic nerve and retinal nerve fiber layer is critical. The progression of damage to the optic disc and retina are an extremely reliable predictor of glaucoma-related functional impairment. Other ocular structures, including the scleral spur, also play a role in the development of glaucoma. Nevertheless, a few patients present with glaucomatous change, and a mere structural assessment fails to provide a sufficient diagnosis [15]. The advancement of imaging devices has facilitated improved visualization of the ganglion cell layer, nerve fiber layer, and optic disk head as potential diagnostic biomarkers. And using a combination of different parameters, structural and functional exams possess the capacity to enhance the early recognition and diagnosis of glaucoma. A summary of the biomarkers and their utility for glaucoma diagnosis are shown in Table 1.

### 3.1. Anterior Segment

#### Scleral Spur Length

Prior research has demonstrated that most of the resistance to the aqueous outflow is situated within the internal region of Schlemm’s canal (SC) [16] and the scleral spur may also play a significant role in maintaining the diameter of the SC lumen. To maintain the SC lumen, the ciliary muscle’s force makes the scleral spur displace backward thus stretching the trabecular meshwork and inner wall of the SC and widening the lumen [17,18]. An additional finding was that the average length of the scleral spur was notably reduced in eyes with POAG when compared to healthy eyes of the same age [19]. This suggests that a shorter scleral spur could potentially serve as a risk factor in the advancement of POAG, as it would lack the capacity to sustain the lumen of SC. Mu et al. reported using swept-source optical coherence tomography (SS-OCT) to conduct observations and make comparisons between the SC and scleral spur length of POAG and healthy individual eyes [20]. The study revealed a significantly shorter scleral spur length of the POAG eyes than the healthy eyes, and also a narrowing scleral spur opening in the POAG eyes. The length of the scleral spur demonstrated a strong diagnostic capacity in distinguishing eyes with primary open-angle glaucoma (POAG) from healthy eyes [20]. Meanwhile, there were other variables that might potentially contribute to the increase in intraocular pressure (IOP) associated with glaucoma. Therefore, it cannot be attributed only to the SC and scleral spur.

### 3.2. Optical Coherence Tomography (OCT)

Optical coherence tomography (OCT) imaging is crucial for the detection and management of glaucoma [21,22,23]. OCT enables precise and non-invasive measurement of alterations in important eye structures related to the diagnosis and the disease nature of glaucoma, ranging from the front part of the eye like the anterior chamber angle to the back part, which includes the macula area, optic nerve head (ONH) and retinal nerve fiber layer (RNFL) [24]. Innovative high-resolution imaging instruments have been developed, leading to notable enhancements in scanning speed, decreased acquisition duration, elevated image clarity, enhanced precision in segmentation, and diagnostic algorithms. These advancements have led to more precise and consistent measures for early detection and better surveillance of glaucoma [25]. Furthermore, OCT technical advancement has significantly improved the visibility of deeper structures within the ONH, which are considered crucial in understanding glaucoma etiology. 

Among these supplementary imaging instruments, spectral-domain optical coherence tomography (SD-OCT) and swept-source OCT (SS-OCT) tend to be the most frequently implemented. Assessing the peripapillary RNFL thickness, optic nerve head, and macular ganglion cell–inner plexiform layer (GCIPL), SD-OCT permits clinicians to objectively and precisely monitor RGCs, as well as their axons and dendrites [25,26]. SS-OCT allows for a wider area in a single image, providing better imaging of the outermost temporal boundary of RNFL defects and brings the utility of SS-OCT wide-field RNFL mapping for early identification of glaucoma. Improved imaging and quantitative assessment of the lamina cribrosa (LC) structural alterations brought on by glaucomatous damage can also be provided by SS-OCT [24].

OCT angiography (OCTA) is a new method that allows for noninvasive and detailed imaging of the small blood vessels in the retina, choroid, and optic disc area. It also offers measurements of the amount of blood vessels in each layer, known as vascular density. Previous research has shown a relation between glaucoma and the flow of blood in the eyes, as well as the disparity between diastolic blood pressure and intraocular pressure (IOP). This discrepancy is linked to a higher occurrence of glaucoma [27,28,29,30,31,32,33,34,35,36].

#### 3.2.1. Segmented Inner Retinal Layer Thickness

Numerous studies have indicated the potential utility of circumpapillary retinal nerve fiber layer (cpRNFL) measurements in the early detection of glaucoma [37]. Nonetheless, it is crucial to note that the depth of cpRNFL can be affected by individual variations in optic nerve head (ONH) structure, such as an oval-shaped and obliquely rotated ONH and peripapillary atrophy, which are commonly observed in individuals with high levels of myopia. However, when there is no concomitant disease, macular parameters are superior in producing more consonant pictures with less variance in structure between people [38]. Kim EK et al. reported that the RNFL, ganglion cell layer (GCL), and inner plexiform layer (IPL) changed as glaucoma progressed, which demonstrates that in individuals with glaucoma, the RGC experiences many dendritic (IPL) and soma (GCL) alterations and RGC axon damage induces rapid pathological alterations in RGC dendrites [4,39]. Slightly in advance of axonal thinning or soma shrinking, morphological alterations in RGC dendrites might be seen in pre-perimetric and early glaucoma [40]. These findings indicate that the measurement of IPL thickness might be a biomarker to detect impairment of RGC function in early glaucoma. Considering the structure–function correlation in segmented layers, Kim’s study observed that IPL and GCIPL thickness have a stronger relationship than RNFL and GCL. And the confidence interval of IPL thickness over the disease progression is narrow. Therefore, measuring IPL thickness along with other segmented inner retinal layer thicknesses may be useful for the early diagnosis and monitoring of glaucoma [4]. Kouros et al. observed that global RNFL thickness had better performance in early glaucoma detection than GCIPL thickness, but sector GCIPL thickness measurements (inferotemperal and minimum GCIPL) had similar performance in sector RNFL thickness (inferior) [41]. Also, an asymmetry finding on the GCIPL thickness maps, the hemifield difference across the horizontal raphe, demonstrated a good early glaucoma detection ability for the GCIPL Hemifield Test [42,43].

#### 3.2.2. Vessel Density, Flow Index, and Foveal Avascular Zone Parameters

The mobility of red blood cells inside blood arteries is the primary source of the signal fluctuation that OCTA detects between images [34]. The formation of a three-dimensional representation of the kinetic contrast resulting from the flow of blood is achieved through the acquisition of many photographs within a brief timeframe, which allows for the viewing and partial measurement of microvascular perfusion. One thing that should be brought to everyone’s attention is the fact that every OCT device that is now on the market detects, represents, and analyzes OCTA signals and microvascular perfusion using a distinct algorithm [35]. Consequently, the results from various OCTA devices might not be directly comparable. However, most of the results can be applied to practical instruments currently on the market [44].

In the OCTA optic disc scan, pre-perimetric glaucomatous eyes was found to have a significant reduction in vessel density in the entire disc area, temporal region of the disc, and in the peripapillary area. And the flow index of these areas showed a considerable decrease compared to healthy individuals [45,46,47]. In the patients with unilateral perimetric glaucoma, the peripapillary and inferotemporal capillary beds were significantly decreased compared to the unaffected eye and in healthy individuals [48,49,50,51]. Kumar et al. reported that superior and temporal sectors of the OCTA images showed more vessel density decrease in glaucoma eyes then healthy eyes [52]. And the asymmetry of vascular density in bilateral eyes, measured by 4.5 × 4.5 mm disc-centered whole-image optic nerve head scans, distinguished healthy people from those who may have glaucoma [53]. The majority of research found a strong connection between the amount of vascular density loss and the severity of glaucoma. Hence, OCTA can identify decreases in blood flow in the ONH before any visible impairment to the visual field occurs. This indicates that OCTA could be valuable for early identification of glaucoma and assessing the risk of glaucoma development.

In the macular OCTA scans, the vessel density decrease is more noticeable in the inferior macular region and superficial vascular plexus layer (internal limiting membrane to inner plexiform layer)—which is now the most often employed OCTA parameter—than in the deep retinal layer because of the projection artifacts from the superficial plexus, and the wider field of the 6 × 6 mm scans centered on the fovea has a higher sensitivity in identifying changes in glaucoma in patients than the 3 × 3 mm scans [29,54,55,56,57,58]. A study using macular whole image vessel density found that a 0.11 μm/year faster decreasing rate of RNFL was associated with every 1% loss of macular vessel density [59]. Several studies also found a significant decrease of vessel density and flow density in deep retinal vascular plexus and the whole retina, and the diagnostic ability of glaucoma of the vessel density in the GCIPL might be better than in the superficial vascular plexus [49,60,61,62,63,64,65]. However, still some studies found that macular vessel density performed no better than OCT GCC thickness in distinguishing early glaucoma from healthy eyes [66,67,68,69]. Choi et al. reported that foveal avascular zone (FAZ)-related parameters (perimeter and circularity index) had a diagnostic value for discriminating glaucoma from healthy subjects. The circularity index would decrease once the FAZ did not have a purely circular shape owing to the progression of deterioration of the capillary network in the parafoveal region. The FAZ perimeter was higher and the circularity index was lower in the POAG eyes. Compared with GCIPL and RNFL thickness, the FAZ-related parameters have similar performance in distinguishing normal and glaucoma eyes [60].

#### 3.2.3. Bruch’s Membrane Opening–Minimum Rim Width (BMO-MRW) and Minimum Rim Area (MRA)

The clinically identified optic disc margin for the neuroretinal rim assessment has no solid anatomic foundation for two reasons: due to the invisible extensions of the Bruch’s membrane (BM) within the disc margin from the image, and the optic nerve head’s (ONH) rim tissue orientation not being traceable. The BM opening–minimum rim width (BMO-MRW) is a parameter that measures the length from the inner limiting membrane to its real anatomical outer border, BMO. The advantages of BMO-MRW include that it considers the expansions of BM that are clinically imperceptible but discovered by SD-OCT. Similar to current methods for measuring peripapillary retinal nerve fiber layer thickness, BMO-MRW measurement considers the varying path of axons across the location of measurement since it is made perpendicular to the axis of the neural tissue [70,71]. Chauhan et al. reported that compared to RNFL thickness, BMO-MRW produced better diagnostic results for glaucoma with current confocal scanning laser tomography (CSLT) or SD-OCT based ONH and RNFLT parameters, excluding the superiornasal quadrant, which exhibited a greater sensitivity to RNFL thickness [71,72,73]. Jonas et al. reported that RNFL thickness and BMO-MRW have comparable areas under the receiver operating characteristic curves (AUROCs) in distinguishing perimetric glaucoma eyes to normal eyes and both showed lower AUROCs in the pre-perimetric glaucoma group. If the specificity was fixed in 95% vs. 90%, RNFL thickness had a sensitivity of 84% vs. 84% and BMO-MRW had a sensitivity of 52% vs. 88% in distinguishing perimetric glaucoma to normal eyes. The BMO-MRW might be influenced by the retinal blood vessels since they may enter the ONH irregularly [74]. Moreover, when it comes to a larger disc, which might have a thinner BMO-MRW in general, a two-dimensional parameter, BMO–minimum rim area (MRA), might have a better diagnostic capability. Introduced by Gardiner et al., BMO-MRA was calculated using the total area of 48 trapeziums, each reaching the inner limiting membrane at an angle above the BMO plane from an identified BMO point [75]. As a result, when comparing different disc sizes, the BMO-MRA should be more beneficial than the BMO-MRW [76,77,78].

#### 3.2.4. Lamina Cribrosa Morphology

The lamina cribrosa is a structure located deep within the eye, specifically within the optic nerve head. It is essentially a sieve-like structure made up of collagen fibers through which the retinal ganglion cell axons pass as they exit the eye to form the optic nerve. These axons transmit visual information from the retina to the brain and were thought to be vulnerable to the pressure gradient stress [79,80]. Over time, this pressure can lead to structural changes in the lamina cribrosa, such as thinning or deformation, which can impede the flow of nutrients to the optic nerve cells and cause damage to the nerve fibers themselves. Previous experimental studies suggested that the morphological changes of the lamina cribrosa precede the thinning of RNFL and defects of the visual field, which means the structural change of the lamina cribrosa could be found in the earliest stage of glaucoma [81,82]. Advanced image technologies including SS-OCT, enhanced depth imaging OCT (EDI-OCT), and adaptive optics -OCT, or -scanning laser ophthalmoscopy (SLO), improved the ability to evaluate the lamina cribrosa.

The lamina cribrosa thickness significantly influences the biomechanics of the optic nerve head (ONH), playing a crucial role in glaucomatous optic nerve change. Studies have shown that the mean laminar thickness was significantly thinner in glaucoma groups (215.41 ± 38.96 µm) than in the control groups (349.08 ± 23.34 µm). And the diagnostic value (as the area under the receiver operating characteristic curve) of the lamina cribrosa thickness for detecting POAG and NTG (0.941, 0.981) was slightly higher than the diagnostic performance of RNFL thickness measurement (0.928, 0.941) [83]. Besides the thinning of lamina cribrosa, posterior bowing, also posterior displacement of the laminar insertion, precedes the RNFL thinning in glaucoma patients. Another parameter which describes the morphology, the lamina cribrosa curvature index (LCCI), may help in distinguishing the glaucomatous optic neuropathy from the normal group and other neuropathies. It is measured by dividing the lamina cribrosa curve depth (LCCD) by the width of the anterior LC surface line and multiplying by 100 (introduced by Seung et al.) [84]. Jeong et al. reported that the greater the LCCI, the faster the RNFL loss rate [79]. The normal lamina cribrosa has a curve, that is, only when the posterior bowing surpasses a certain threshold will the optic nerve axons get injured. Seung et al. suggested the threshold of LCCI around 9.51 though it may differ among individuals and requires further refinement in a future investigation of greater magnitude.

### 3.3. Magnetic Resonance Imaging (MRI)

As a neurodegenerative disease, glaucoma causes damage not only to the retinal ganglion cells but also their dendrites and axons, and involves damage along the visual pathways to the brain, such as the optic tract, lateral geniculate nucleus (LGN), optic radiation, and visual cortex [85,86,87,88,89,90]. Recent studies have focused on the MRI utility of assessing the glaucomatous injuries within the brain, including atrophy and degeneration of the visual cortex and visual pathway, and the diffusion tensor imaging (DTI)-derived parameter, fractional anisotropy (FA) [91,92].

#### 3.3.1. Morphometry

Anatomical magnetic resonance imaging (MRI) offers comprehensive details on the morphological characteristics of different brain areas, often pertaining to size and shape. With the advent of MRI scanners with field strengths of 3 Tesla or more, researchers can now produce images of exceptional quality that clearly distinguish different brain tissues and allow them to examine the relationships between different brain structures and biological, psychological, and clinical parameters. Morphometry is the quantitative measurement of a structure’s dimensions and forms [93].

In a series of studies investigating the effects of glaucoma on the brain, several key findings were observed. Using voxel-based techniques, Bogorodzki et al. revealed that individuals with a loss of vision in one eye only due to advanced open-angle glaucoma exhibited a noticeable reduction in the thickness of the visual cortex compared to a normal age-matched group [94]. Another study using high-precision magnetic resonance imaging revealed a reduction in grey matter volume in patients with advanced glaucoma in a number of brain regions, including the lingual, calcarine, postcentral gyrus, inferior frontal, superior frontal gyrus, and Rolandic operculum, and also in the right cuneus, right inferior occipital gyrus, right supramarginal gyrus, and left paracentral lobule [95]. The findings collectively suggest that glaucoma can lead to structural changes in the brain, particularly in regions associated with vision processing. These changes may vary depending on the stage of the disease. Additionally, the cortical thickness of the visual cortex may serve as a potential diagnostic marker for glaucoma, especially when considering age-related changes in cortical thickness. Rodolfo et al. revealed that in an early glaucoma patient, a thinning cortex was majorly found in the primary visual cortex in MRI and the RNFL and GCL/IPL complex also showed morphological defects on OCT while the visual field exam still remained normal [96]. Early detection and monitoring of these brain changes through techniques like MRI could aid in the diagnosis and management of glaucoma.

#### 3.3.2. Fractional Anisotropy (FA) Values and Mean Diffusivity (MD) of DTI

Postmortem examinations have shown the presence of glaucomatous neuronal degeneration in all regions of the central visual pathways, resulting in significant visual field impairments in both eyes. The quantitative measurement of anterior visual pathway compression may be achieved by the utilization of diffusion tensor imaging (DTI), which employs fractional anisotropy (FA) and mean diffusivity (MD). DTI measures the diffusion of water molecules in tissues, particularly in the brain’s white matter tracts. It provides information about the microstructural organization and integrity of white matter fibers by analyzing the directionality and magnitude of water diffusion [97]. FA is a scalar value derived from the diffusion tensor, representing the degree of anisotropy within a voxel [98]. High FA values indicate highly directional diffusion, typically found in intact white matter tracts, while low FA values suggest disrupted or disorganized tissue structure. A drop in FA and an increase in MD may suggest the presence of structural impairment in the optic nerve axon among individuals diagnosed with glaucoma [99].

Prior research has demonstrated that individuals with glaucoma have notably elevated MD and reduced FA in relation to the optic nerve and optic radiation. These findings are consistent with the severity of glaucoma, as well as the morphological alterations observed in the optic nerve head and retinal nerve fiber layer [100,101,102,103]. The MD values exhibited a greater magnitude at the proximal location of the optic nerve head in comparison to the distal location. In contrast, a reduction in FA was seen solely in relation to the stage of the patient, regardless of the location of the optic nerve. Furthermore, in the early stage of glaucoma, there was a noticeable rise in comprehensive diffusivities at the proximal location. Conversely, at the distal location, a decrease was observed in the diffusivity that was the highest, whereas there was a rise in the diffusivities that were intermediate and the smallest. The results indicate that DTI, which has a high sensitivity for FA and a high specificity for MD, might be a useful additional diagnostic technique for evaluating structural changes in retinal ganglion cells and optic nerves in glaucoma [96,104].

## 4. Conclusions

Glaucoma may not even have any symptoms in the early stages and does not cause much restriction in daily life because the preservation of central vision comes at the expense of peripheral vision, which is more affected. It is only in the late stages, when significant and irreversible loss of vision has occurred causing weakened spatial perception and difficulties in certain daily activities, that it may be noted and diagnosed. Therefore, early diagnosis should be taken promptly.

The structural changes include the shortening scleral spur length, decreasing GCL/IPL thickness, BMO-MRW/MRA, decreasing vessel density/flow density, FAZ parameters, alteration of lamina cribrosa morphology, and the neurodegeneration noted from MRI. Relying merely on a single biomarker may not adequately assess glaucomatous changes, but combining multiple biomarkers and functional tests increases the sensitivity and specificity of early glaucoma diagnosis. Further investigation is warranted to examine the diagnostic and prognostic significance of these biomarkers combined with others, including perimetry and electrophysiological tests.

## Figures and Tables

**Table 1:** Summary of the biomarkers and their utility for glaucoma diagnosis.

Structural Biomarkers	Findings	Limitations	Utility for Glaucoma Detection
Scleral spur length	Shorter scleral spur length of POAG eyes than the healthy	The increase of IOP cannot be attributed only to the Schlemm’s canal and scleral spur	Low
GCL/IPL thickness *	Decreased IPL and GCIPL thickness	Still may be affected by highly myopic eyes (GCIPL hemifield test provides a superior diagnostic ability)	High
	Less affected by the degree of myopia and myopia-related optic disc change than RNFL thickness		
Vessel density and flow index	Decrease of vessel density and flow density in deep retinal vascular plexus and the whole retina	Superficial layer of retinal vasculature to obscure the deeper vessels of the retina Artifacts Ocular vascular changes in specific conditions including smoking, cardiovascular disease, hypoxia, and hyperoxia	Moderate
FAZ-related parameters * (perimeter and circularity index)	Higher FAZ perimeter Lower circularity index		Low
BMO-MRW *	Better determination of the borders of the neuroretinal rim Useful in myopic eyes	Affected by the diversity of disc size and retinal blood vessels	Moderate
BMO-MRA *	Useful in different disc size	Might not reflect the actual minimum area	
Lamina cribrosa morphology	Decreased laminar thickness Posterior displacement of the laminar insertion Greater lamina cribrosa curvature index	Need for prospective studies evaluating lamina cribrosa changes over time	Low–Moderate
Cortical thickness of the visual cortex	Thinning cortex was majorly found in the primary visual cortex	High cost; time consuming	Low
Fractional anisotropy (FA) values and mean diffusivity (MD)	Elevated MD and reduced FA in relation to the optic nerve and optic radiation	High cost; time consuming	Low

* GCL/IPL = ganglion cell layer (GCL) and inner plexiform layer (IPL). * FAZ = foveal avascular zone. * BMO-MRW/MRA = Bruch’s membrane opening–minimum rim width (BMO-MRW) and minimum rim area (MRA).
